# Cutaneous Autonomic Pilomotor Testing to Unveil the Role of Neuropathy Progression in Early Parkinson’s Disease (CAPTURE PD): Protocol for a Multicenter Study

**DOI:** 10.3389/fneur.2017.00212

**Published:** 2017-05-26

**Authors:** Timo Siepmann, Alexandra Pintér, Sylvia J. Buchmann, Leonie Stibal, Martin Arndt, Anne Sophie Kubasch, Marie Luise Kubasch, Ana Isabel Penzlin, Elka Frenz, Wagner Zago, Tamás Horváth, Szabolcs Szatmári, Dániel Bereczki, Annamária Takáts, Tjalf Ziemssen, Axel Lipp, Roy Freeman, Heinz Reichmann, Kristian Barlinn, Ben Min-Woo Illigens

**Affiliations:** ^1^Department of Neurology, University Hospital Carl Gustav Carus, Technische Universität Dresden, Dresden, Germany; ^2^Department of Family Medicine, Semmelweis University, Budapest, Hungary; ^3^Department of Neurology, Beth Israel Deaconess Medical Center, Harvard Medical School, Boston, MA, United States; ^4^Department of Neurology, Charite University Medicine Berlin, Berlin, Germany; ^5^Center for Rare Diseases, University Hospital Carl Gustav Carus, Technische Universität Dresden, Dresden, Germany; ^6^Prothena Biosciences Inc., Portland, OR, United States; ^7^Department of Hydrodynamic Systems, Budapest University of Technology and Economics, Budapest, Hungary; ^8^Department of Neurology, Semmelweis University, Budapest, Hungary

**Keywords:** autonomic, Parkinson’s disease, diagnosis, pilomotor, axon-reflex

## Abstract

**Background:**

In Parkinson’s disease (PD), alpha-synuclein accumulation in cutaneous autonomic pilomotor and sudomotor nerve fibers has been linked to autonomic nervous system disturbances even in the early stages of the disease. This study aims to assess the association between alpha-synuclein-mediated structural autonomic nerve fiber damage and function in PD, elucidate the role of neuropathy progression during the early disease stages, and test reproducibility and external validity of pilomotor function assessment using quantitative pilomotor axon-reflex test and sudomotor function *via* quantitative direct and indirect test of sudomotor function.

**Methods/design:**

A prospective controlled study will be conducted at four study sites in Europe and the USA. Fifty-two male and female patients with idiopathic PD (Hoehn and Yahr 1–2) and 52 age- and sex-matched healthy controls will be recruited. Axon-reflex-mediated pilomotor erection will be induced by iontophoresis of phenylephrine on the dorsal forearm. Silicone impressions of the response will be obtained, scanned, and quantified for pilomotor muscle impressions by number, impression size, and area of axon-reflex spread. Axon-reflex-mediated sweating following acetylcholine iontophoresis will be quantified for number and size of droplets and axon-reflex spread. Sympathetic skin responses, autonomic and motor symptoms will be evaluated. Tests will be performed at baseline, after 2 weeks, 1, 2, and 3 years. Skin biopsies will be obtained at baseline and after 3 years and will be analyzed for nerve fiber density and alpha-synuclein accumulation.

**Discussion:**

We anticipate that progression of autonomic nerve dysfunction assessed *via* pilomotor and sudomotor axon-reflex tests is related to progression of autonomic symptom severity and alpha-synuclein deposition. Potential applications of the techniques include interventional studies evaluating disease-modifying approaches and clinical assessment of autonomic dysfunction in patients with PD.

**Clinical trail registration:**

TRN NCT03043768.

## Introduction

### Background

In patients with Parkinson’s disease (PD), early diagnosis enables timely symptomatic treatment and improvement of quality of life (1). However, diagnosis of prodromal and early disease stages is challenging since only few diagnostic techniques are available (2). Symptoms due to dysregulation of autonomic nervous system functions such as impaired thermoregulation and cardiovascular symptoms are prevalent in patients with PD. In fact, these symptoms frequently precede onset of motor symptoms and reduce quality of life (2). The pathophysiological mechanisms leading to autonomic disturbances in patients with PD disease are not fully understood. However, there is accumulating evidence of alpha-synuclein-mediated damage to autonomic postganglionic adrenergic and cholinergic skin nerve fibers (3–5). A recent paper of Wang and colleagues showed that non-specific nerve fiber stain protein gene product 9.5 (PGP) in epidermal biopsies is reduced whereas the number of alpha synuclein positive fibers is increased in PD patients compared to healthy subjects (6). This study also showed that the alphasynuclein/PGP ratio is increased in cutaneous sudomotor and pilomotor fibers in PD patients and that higher alpha-synuclein/PGP ratios are associated with more severe autonomic dysfunction and more advanced motor symptoms. Importantly, a clear increase in pathology was seen between Hoehn and Yahr 1 and 2, suggesting a possible utility in relatively short clinical studies. Although it may be concluded that quantification of alpha-synuclein deposition in pilomotor muscles is a valid biomarker for PD, this technique is limited by its invasive nature and by the uncertainty of whether the alpha-synuclein staining detected in the nerves is, indeed, the cause of the observed symptomatic functional deficits.

Autonomic skin nerve fibers can not only be quantitatively assessed for structural damage but also function. In fact, functional assessment of autonomic skin nerves has taken on increasing importance in the evaluation of autonomic neuropathy (7–9). Cutaneous unmyelinated C-fibers and lightly myelinated A-delta fibers (small fibers) are responsive to a range of physical, chemical, and mechanical stimuli that can induce an axon-reflex mediated response. The axon-reflex was first described in vasomotor small fibers. Activation of these postganglionic cutaneous fibers results in orthodromic conduction to the spinal cord and antidromic conduction to other axon branches. On activation, both C-fibers and A-delta fibers release SP and CGRP leading to vasodilation and plasma extravasation (10–12). This neurally mediated response can be evoked by chemical (e.g., acetylcholine, histamine and capsaicin) and electrical stimulation (13, 14).

While quantification of vasomotor axon-reflex responses for diagnostic purposes is limited by interindividual variability, further developments focused on postganglionic small sudomotor fibers. The acetylcholine-induced sudomotor C-fiber axon-reflex also involves the stimulation of postganglionic sympathetic nerve terminals. Conversely to the vasomotor axon-reflex, an antidromic impulse travels to a branch point and then returns orthodromically to release acetylcholine from a neighboring nerve terminal. Acetylcholine diffuses across the neuroglandular junction and binds to muscarinic receptors on the eccrine sweat glands and evokes a sweat response (8, 15, 16). The sudomotor axon-reflex can be quantified by the quantitative sudomotor axon-reflex test (QSART) and the quantitative direct and indirect test of sudomotor function (QDIRT) (16, 17). QSART assesses axon-reflex-mediated sweating with temporal resolution but is limited by demanding technical settings and absence of spatial resolution. QDIRT quantifies axon-reflex-mediated sweating with both temporal and spatial resolution and requires less demanding technical setting (15). The role of small fiber-mediated sudomotor dysfunction in PD is poorly elucidated. Several studies indicated impairment of sudomotor function even in early disease stages when evaluated *via* sympathetic skin response (SSR), a technique which assesses skin conductance levels (SCLs) following sympathetic stimulation with high sensitivity but is limited by high interindividual variability and the absence of differentiation between pre-and postganglionic function (18–20). However, another study of SSR showed not such impairment in PD patients without autonomic symptoms, suggesting that the technique is not sufficient for exploration of subclinical autonomic dysfunction in early PD (21). Another approach to quantify small fiber neuropathy is the skin wrinkling test, which includes hand immersion in heated saline and subsequent visual grading of skin wrinkling. The technique has been used in patients with PD and Parkinsonism, but this study was limited by the absence of healthy controls. The technique is easy to use and inexpensive. However in this study, it remained unclear to what degree observed differences in wrinkling of the skin did reflect postganglionic small nerve fiber function rather than large fiber integrity limiting interpretability of the results (22).

Only few data are available on postganglionic small fiber function in PD and, to date, there are no studies of sudomotor axon-reflex response with temporal and spatial resolution in this prevalent synucleinopathy (23). Whereas the sudomotor axon-reflex response is a postganglionic cholinergic function, pilomotor erection is executed *via* postganglionic noradrenergic fibers (17, 24).

This multicenter protocol is based on previous studies of pilomotor erection that have been undertaken by two of the study groups involved in the design of the present protocol. First, we demonstrated that iontophoretic stimulation of cutaneous adrenergic small fibers with phenylephrine elicits axon-reflex-mediated pilomotor erection (goose bumps) in an indirect region surrounding the area of direct stimulation, which can be evaluated using the Quantitative Pilomotor Axon-Reflex Test (QPART) (24). In another investigation of pilomotor function in patients with PD versus healthy control subjects, we showed that functional integrity of pilomotor nerve fibers is impaired in early stages of PD and correlates negatively with severity of autonomic symptoms (25). Taken together, the data deriving from our previous investigations indicate that pilomotor axon-reflex assessment might be useful in the investigation of disease-related pathology and supplement other clinical markers of autonomic neuropathy in PD. However, the specific role or pilomotor and sudomotor nerve fiber damage in PD remains to be elucidated.

### Specific Aims

With the goal of developing novel endpoints for early stage trials of disease-modifying therapies in PD, specific aim of this study is to assess reproducibility, external validity of QPART and QDIRT in the evaluation of patients with PD. Moreover, this study aims to assess the hypothesis that functional impairment in pilomotor and sudomotor nerve fibers in early PD relates to the extent of alpha-synuclein-mediated structural damage to these fibers and may, therefore, constitute a potential non-invasive biomarker for autonomic neuropathy in PD.

### Protocol Outline

This study is designed as a longitudinal, controlled, blinded, diagnostic multicenter study, and will be conducted at four study sites in Dresden, Germany (Department of Neurology, University Hospital Carl Gustav Carus, coordinating site), Boston, USA (Department of Neurology Beth Israel Deaconess Medical Center, core lab for data analysis), Budapest, Hungary (Department of Neurology, Semmelweis University) and Berlin, Germany (Department of Neurology, Charite University Medicine Berlin).

Patients with early PD and age and gender-matched healthy control subjects will be longitudinally evaluated for functional and structural damage to pilomotor and sudomotor skin nerve fibers over a period of 3 years. SSRs will be evaluated as comparative measure of sudomotor function. Details on the timeline and applied study techniques are listed in the Section “Stepwise Procedures” (paragraph 3).

## Materials and Equipment

The study procedures applied in this protocol will employ equipment and materials as listed below separately for each study procedure.

### Quantitative Pilomotor Axon-Reflex Test

Materials and equipment used for the QPART include an iontophoresis stimulation box (Phoresor II-Auto-PM850; IOMED Inc., Salt Lake City, UT, USA), disposable drug delivery capsule electrodes (LI-611; Perimed^®^, Järfälla, Sweden), 0.01% phenylephrine solution, silicone-based two-phase material (Silasoft^®^; Microsonic Inc., Ambridge, PA, USA), a high resolution scanner connected to a personal computer equipped with image analyzing software (Fuel3D^®^ Studio Starter; Fuel 3D^®^ Technologies Limited, Chinnor, UK).

### Quantitative Direct and Indirect Test of Sudomotor Function

Materials and equipment used for the QDIRT include an iontophoresis stimulation box (Phoresor II-Auto-PM850; iOMED Inc., Salt Lake City, UT, USA), disposable drug delivery capsule electrodes (LI-611; Perimed^®^, Järfälla, Sweden), 10% acetylcholine solution, indicator dye consisting of providine iodine and corn starch, an 18 Megapixel Camera (EOS 60D; Canon Inc., Tokyo, Japan), with 100-mm macro lens, a personal computer equipped with image analyzing software (Image Pro^®^ Plus 6.0; Media Cybernetics Inc., Rockville, MD, USA).

### Sympathetic Skin Response

Sympathetic Skin Response is measured using a polygraph (PowerLab; ADInstruments, Bella Vista, NSW, Australia).

## Stepwise Procedures

### List of Stepwise Study Procedures

Subjects will be assessed for eligibility by a listed study physician. Eligible subjects will be informed in detail about the study protocol and each applied study procedure as well as the risks of each procedure. Oral and written informed consent will be obtained from each subject. At baseline, all subjects will undergo medical history, physical examination; specific study procedures as described in the Section “Study Techniques.” These testing procedures will be repeated, after 1–2 weeks, 1, 2, and 3 years post-baseline. All testing will be performed in a temperature and humidity controlled environment. Only at one site (Dresden, Germany), subjects will additionally undergo the skin punch biopsy procedure as described above at baseline, after 1 and 3 years. The timeline of this study is illustrated in detail in Table 1.

**Table 1:** **A table of stepwise study procedures in chronological order**.

Procedure	Baseline	1–2 weeks	12 months	24 months	36 months
Medical history, physical examination	x				
Quantitative pilomotor axon-reflex test	x	x	x	x	x
Sympathetic skin response	x	x	x	x	x
Quantitative direct and indirect test of sudomotor function	x	x	x	x	x
UPDRS	x	x	x	x	x
SCOPA-AUT	x	x	x	x	x
Biopsies (only at one site)	x		x		x

### Selection of Subjects

Thirteen patients with PD and 13 healthy control subjects will be recruited at each participating site. Patients will be recruited from university hospital-based PD outpatient clinics at each site. Control subjects will be recruited through local advertising at university hospitals. Male and female PD patients [idiopathic parkinsonism, Hoehn and Yahr (H and Y) scores 1–2] aged 35–80 years with mild motor symptoms (Hoehn and Yahr 1–2) and age- and gender-matched healthy control subjects will be recruited at each site. We will exclude subjects who fulfill with one or more of the following criteria: any dermatological disorders affecting the cutaneous testing regions, treatment with tricyclic antidepressants, noradrenergic antidepressants, beta-blockers, alpha-adrenergic agonists or antagonists, cholinergic or anticholinergic agents, known allergy to phenylephrine or acetylcholine, elevated alcohol consumption (more than four alcoholic beverages/week), nicotine consumption within the past 5 years, known disorders affecting autonomic functions (including diabetes, pure autonomic failure, inflammatory demyelinating polyradiculoneuropathies, multiple system atrophy, atypical Parkinson syndromes, body mass index over 25 kg/m^2^, acute or chronic renal disease, gout, rheumatoid arthritis, Lupus, Sjögren’s syndrome, Triple-A syndrome, autonomic neuropathies not related to PD). Additional exclusion criteria for healthy control subjects include any acute or chronic disease and chronic intake of medication. All subjects will be asked to avoid caffeine on the days of testing. Missing data due to missed follow-up visits will be minimized by monitoring of adherence as well as phone calls and e-mails prior to each follow-up.

## Methods and Anticipated Results

### Study Techniques

#### Quantitative Pilomotor Axon-Reflex Test

**Figure 1:**
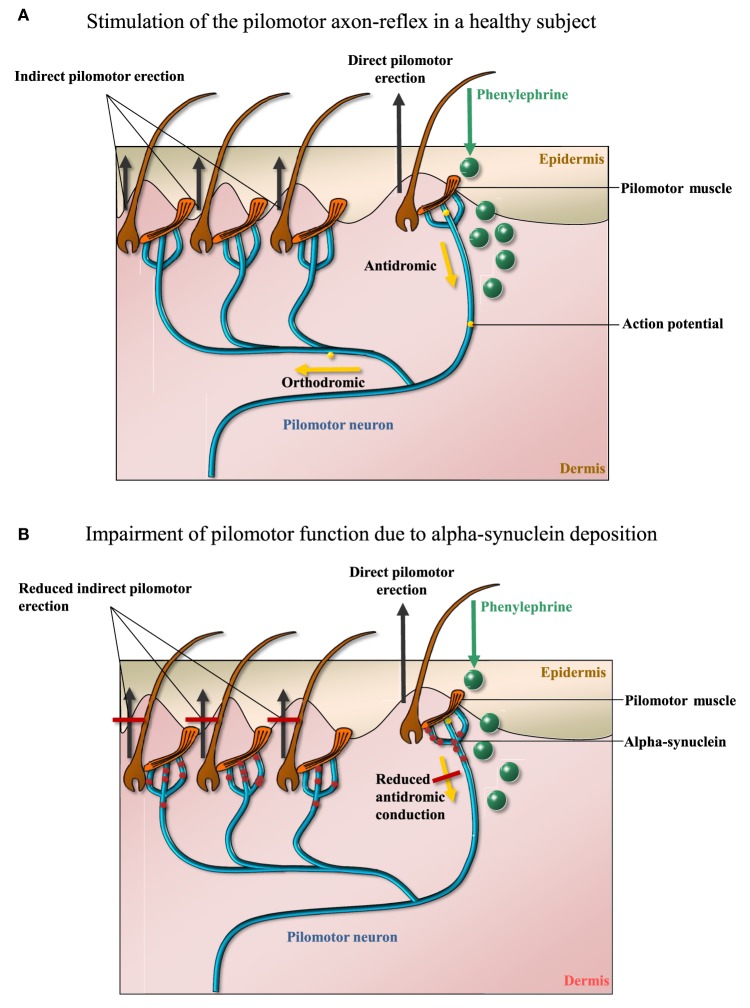
**(A)** The mechanism whereby iontophoresis of phenylephrine induces a local neurogenic pilomotor erection (“goose bumps”). Receptor-mediated pilomotor erection is provoked in the cutaneous area of application (direct response), an action potential travels antidromically and then orthodromically to neighboring pilomotor muscles. Thus, pilomotor erection is evoked in an area outside the area of phenylephrine application (indirect response). Quantification of indirect pilomotor erection is a measure of functional integrity of pilomotor nerve fibers. **(B)** Reduced axon-reflex-mediated pilomotor erection due to alpha-synuclein-mediated damage to the pilomotor nerve fiber (m: months, w: weeks). The figures were designed by TS and BMI.

**Figure 2:**
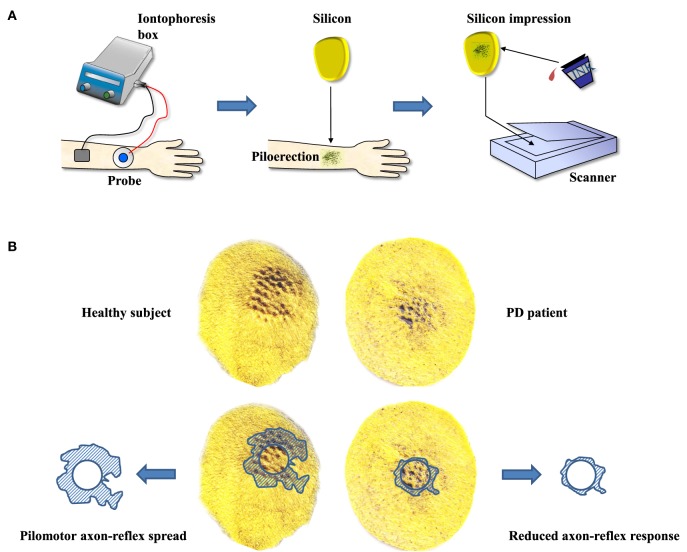
**(A)** The stepwise procedures of evoking and quantifying pilomotor erection. Iontophoresis of phenylephrine is followed by obtaining silicone impressions of the pilomotor erection response. Impressions are highlighted and scanned. **(B)** Anticipated results (example observation from an unpublished pilot study) of a healthy subject versus a patient with Parkinson’s disease (PD). The comparison of scans illustrates the anticipated reduction of pilomotor axon-reflex spread in PD. The figures were designed by TS and BMI.

Iontophoresis of phenylephrine is used to induce axon-reflex-mediated piloerection as previously described (24). Briefly, a drug delivery capsule electrode (LI-611, Perimed, Sweden) is affixed on the testing area on the dorsal forearm. The inner chamber of this capsule, open to the skin surface, is filled with 0.4 ml of 0.01% phenylephrine solution. The drug delivery electrode is then connected to the iontophoresis stimulation box (Phoresor-PM850, IOMED, USA). Iontophoresis is performed over a 1-cm diameter skin region with 0.5 mA over 5 min. Silicone impressions of piloerection are obtained to create a local topographic map of piloerection. A silicone-based two-phase material with high liquidity (Honigum Light, DMG, Hamburg, Germany) is placed over the skin for 10 s. A dispensing device (Automix dispenser, DMG, Hamburg, Germany) is used to mix and apply both phases of the silicone in order to standardize application of silicone and avoid any mechanic pressure during the application process, thus further reducing sweating. The silicone cures for 5 min has toner applied to mark the pilomotor impressions, excess toner is wiped free, and the silicone is scanned to capture the image digitally. Blinded observers analyze silicone scans using image analyzing software (Image Pro Plus 6.0, Media Cybernetics, Bethesda, MD, USA). Silicone impressions of erect hair follicles are quantified by number and area. The outline of the total area of piloerection is defined as a line connecting the outer edges of the most peripheral erect hair follicle impressions. The indirect area of axon-reflex-mediated piloerection can be calculated by subtracting the area of phenylephrine application from the total area of piloerection. QPART is performed on both forearms and both lower legs. Potential artifacts of the method include confounding sweat droplets. This will be overcome by selecting a silicone material with high liquidity to minimize pressure to the skin as well as a short placement time of 10 s. We anticipate that pilomotor function is impaired in patients with PD compared to healthy subjects, is related to alpha-synuclein-mediated structural damage to pilomotor nerve fibers and progresses over time. The anticipated pathophysiological mechanism of pilomotor erection in healthy subjects and patients with PD is illustrated in Figure 1. Methodology of the silicone impression technique as well as anticipated differences between controls and PD are illustrated in Figure 2.

#### Quantitative Direct and Indirect Test of Sudomotor Function

Axon-reflex-mediated sweating is measured after iontophoresis of 10% acetylcholine by drying the stimulated region on the volar aspect of the forearm and dusting with indicator dye (povidone–iodine and cornstarch) followed by repeated digital photographs taken every 15 s for 7 min (8 Megapixel or higher camera with 100-mm macro lens). The QDIRT images are uploaded as a sequence and analyzed using an automated image analyzing algorithm as previously described (17). Sweat droplets in the axon-reflex region are quantified by number, size, and axon-reflex spread. Spatial spread of the axon-reflex-mediated sweating including the direct area of stimulation is calculated by defining the outline of the total area of sweating as a line connecting the outer edges of the most peripheral droplets. The direct area of acetylcholine application is then subtracted from the total area to calculate the axon-reflex area. QDIRT is performed on both forearms and both lower legs. A limitation of this study technique is that it has not been validated in patients with PD. Therefore, SSRs are also evaluated in this protocol as a less specific but well established comparative measure of sudomotor function. We anticipate that sudomotor function is impaired in patients with PD compared to healthy subjects, is related to alpha-synuclein-mediated structural damage to sudomotor nerve fibers, and progresses over time.

#### Sympathetic Skin Response

Sympathetic skin response is evaluated following sudden deep respiration as previously described (15) Briefly, the SCL is measured in μSiemens (μS) from two medial phalanges (index and third finger) with a Powerlab^®^polygraph (AD Instruments, Bella Vista, NSW, Australia). The maximum increase in amplitude following sudden deep respiration (SSR) is calculated to quantify the functional reactivity of sweat glands. While the technique shows high sensitivity for changes in sweat output, drawbacks include high interindividual variability and the lack of differentiation between pre- and postganglionic sudomotor function (15). Therefore, sudomotor function will also be assessed by QDIRT, a specific measure of postganglionic sudomotor function, which previously showed low variability (17). We anticipate that SSR is reduced in patients with PD compared to healthy control subjects but the difference might be less pronounced than in QDIRT parameters due to high interindividual variability.

#### Skin Biopsies: Alpha-Synuclein/PGP Ratio

Immunhistochemical staining for alpha-synuclein and intraepidermal nerve fibers will be performed in four skin punch biopsies obtained from the QPART testing sites and two control sites (lower legs) as previously described (6). Three-millimeter biopsies will be obtained following local anesthesia with 2% lidocaine. The procedure was shown to be safe and easy to perform (26). Biopsy specimens are fixed in Zamboni solution for 18 h and cryoprotected overnight (20% glycerol and 20% 0.4 M Sorensen buffer). Tissue blocks are fixed in 10% formalin solution over 18–24 h at room temperature, then washed, and stored in 0.01 M PBS solution at 4°C. Blocks are cut using freezing microtome and 20 tissue sections are analyzed. Additional sections are analyzed wherever pilomotor muscles or sweat glands are not identified in the original sections. All specimens are quantified for alpha-synuclein concentrations and pilomotor and sudomotor nerve fiber density by a laboratory experienced in the technique (Prothena Biosciences Laboratory, South San Francisco, CA, USA). Alpha-synuclein/intraepidermal nerve fiber density ratios are calculated for pilomotor muscles and sweat glands. The procedure has been shown safe; it includes not only reversible minimum skin damage and previous studies using the same procedure noted no adverse events (4, 6). Specimens will be preserved for at least 10 years and will be destroyed if the subject withdraws from the study.

Possible pitfalls include damage to the specimen due to incorrect removal of the punch. To address this issue investigators who will operate the procedure (Martin Arndt, Timo Siepmann) are trained by a physician experienced with the technique (BMI). We anticipate that alpha-synuclein/intraepidermal nerve fiber density ratios are increased in pilomotor muscles and sweat glands of patients with PD compared to healthy subjects. We further anticipate that this measure of alpha-synuclein damage increases over time and is related to functional decline of pilomotor and sudomotor nerve fibers.

#### Evaluation of Motor and Autonomic Symptoms

Progression of motor and autonomic symptoms are assessed through validated symptom scores MDS-UPDRS motor part (27) and SCOPA-AUT (28, 29). In order to assure precise grading, investigators performing/evaluating these scales are either physicians with experience in the assessment of patients with PD (Elka Frenz, Timo Siepmann, Kristian Barlinn) or are be trained by these physicians prior to beginning of the study.

### Statistical Considerations

#### Primary Endpoint and Sample Size Calculation

The absolute number of indirect hair follicle indentations is a primary endpoint. The sample size was calculated based on an interim analysis of an unpublished pilot study using results from assessment of 12 PD patients. A SD of 8.2 was found by means of QPART (*p* < 0.05 PD versus healthy controls). Based on this observation, we will be able to detect a difference in means of 2.5 (16%) with 90% power at a significance level of 0.05 by including 13 subjects and 13 PD patients at each participating study site.

#### Statistical Analysis Plan

Analyses are performed using the statistical software package STATA^®^ (Version 14, College Station, TX, USA). Differences between groups are analyzed using ANOVA with repeated measures. *Post hoc t*-tests are performed where appropriate. Pearson’s correlation analyses are performed to assess the relations between disease length, motor symptom progression (UPDRS motor part), perceived autonomic symptoms (SCOPA-AUT), and progression of autonomic small fiber dysfunction. Univariate and multivariate regression models are built to assess whether pilomotor and sudomotor functional measures are independently associated with autonomic and motor symptom severity. All factors that emerge as predictor variables in the univariate analysis at *p* < 0.1 are included in the multivariate model as candidate variables and then removed by stepwise backward selection procedure with removal threshold set at *p* = 0.2. In addition, a forward selection procedure will be performed to confirm the robustness of the multivariate model. A proximal similarity model is built to estimate external validity.

### Adverse Events

All subjects will be questioned for adverse events (undesired harmful effect resulting from the study procedure) after each study procedure. Adverse events are not expected in this study as each applied procedure has minimum risks of adverse events. Serious adverse events (events resulting in death, life-threatening condition, inpatient hospitalization or prolongation of existing hospitalization, persistent or significant disability/incapacity, the necessity of intervention to prevent permanent impairment or damage) are not expected in this study, as each applied procedure has minimum risks of serious adverse events.

### Data Management

All data will be entered into the case report form. The case report form will be signed by the study physician and will be stored for at least 10 years in the local study archive. All data will be pseudonymized and entered into study data base using REDCap (Research Electronic Data Capture, ©2015 Vanderbilt University), a standardized and secure web application for building and managing online surveys and databases.

### Informed Consent

The investigator will obtain informed consent of a subject or his/her designee prior to any study-related procedures. The study will be explained in detail to the patients by the investigators. In addition, each patient will receive detailed study information in writing. After all questions have been answered, the patient will sign the written informed consent form. One original copy of the written informed consent will be kept in the study center and a second original copy will be handed out to the patient.

### Limitations and Potential Pitfalls

This protocol comprises techniques of functional autonomic fiber assessment (QPART and QDIRT) that are not the most widely used methods of their kind, which might reduce comparability with previous research. However, we have specifically chosen these techniques as they allow assessment of axon-reflex responses with both temporal and spatial resolution. We anticipate that this technical feature will help overcome those limitations of axon-reflex assessment that were apparent in previous studies applying more conventional techniques. In particular, we seek to reduce intraindividual and interindividual variability by increasing the dimensions in which the axon-reflex response is evaluated. Another possible pitfall of this protocol originates in the duration of this longitudinal 3-year study. Namely, there is a considerable risk of loss to follow-up due to the relatively long intervals between the study visits. Therefore, we plan to increase protocol adherence by reaching out to our subject prior to each visit both *via* e-mail and telephone.

## Interpretation of Anticipated Results

Demonstrating generalizability and reproducibility of the QPART and the QDIRT technique in a population of PD patients might support the potential utility of these techniques in the early diagnosis and monitoring of the disease. The anticipated association of functional and structural pilomotor and sudomotor nerve fiber impairment might improve our understanding of the role of alpha-synuclein-mediated neuropathy in PD support the capacity of these techniques to assess specific fiber damage. Both QPART and QDIRT might thus provide non-invasive tools for interventional studies evaluating disease-modifying approaches and clinical assessment of autonomic dysfunction in patients with PD.

## Ethics Statement

The study protocol has been approved by the institutional review board (IRB) of Technical University of Dresden (Die Ethikkommission an der TU Dresden, Office for Human Research Protections IDs: IRB00001473, IORG0001076, IRB study reference: EK349082015) as well as by the institutional review board of each participating site (Boston IRB number: 2016P000141, Budapest IRB number: 004513-004/2016/OTIG, Berlin accepts IRB approval from Technical University of Dresden IRB). The study was registered on https://ClinicalTrials.gov (Identifier: NCT030437680).

## Author Contributions

TS has made substantial contributions to conception and design of the work, drafting the work, final approval of the version to be published, and agreement to be accountable for all aspects of the work in ensuring that questions related to the accuracy or integrity of any part of the work are appropriately investigated and resolved. AP, SB, LS, MA, AK, MK, AIP, EF, WZ, TH, SS, DB, AT, TZ, AL, RF, HR, KB, and BI have made substantial contributions to conception and design of the work, revising the work critically for important intellectual content, final approval of the version to be published, and agreement to be accountable for all aspects of the work.

## Conflict of Interest Statement

WZ is a fulltime employee of Prothena Biosciences Inc. The other authors declare no commercial or financial relationships that could be construed as a potential conflict of interest.

## Abbreviations

UPDRS, Unified Parkinson’s Disease Rating Scale; PD, Parkinson’s disease; PGP, protein gene product; QDIRT, quantitative direct and indirect test of sudomotor function; QPART, quantitative pilomotor axon-reflex test; QSART, quantitative sudomotor axon-reflex test; SCOPA-AUT, scale for outcomes in Parkinson’s disease for autonomic symptoms; SSR, sympathetic skin response; SCL, skin conductance level.
